# Post-ICU Outcomes in a Low-Resource Setting: A Cohort Study From Rwanda

**DOI:** 10.1097/CCE.0000000000001399

**Published:** 2026-04-24

**Authors:** Geofrey Bahati, Wellars Niragire, Faustin Niyonshuti, Eric Mugwaneza, Dawit Kebede Huluka, Christian M. Mukwesi

**Affiliations:** 1 All authors: Division of Clinical Services, Department of Anaesthesia and Critical Care, Rwanda Military Teaching Hospital, University of Rwanda, Kigali, Rwanda.

**Keywords:** critical illness survivorship, intensive care unit, low-resource settings, post-intensive care unit outcomes, sub-Saharan Africa

## Abstract

**IMPORTANCE:**

Survivorship after critical illness in low-resource settings is inadequately characterized.

**OBJECTIVES:**

To assess 90-day mortality and multidimensional recovery following ICU discharge.

**DESIGN, SETTING, AND PARTICIPANTS:**

Prospective cohort of adults discharged alive from a national referral ICU in Rwanda.

**MAIN OUTCOMES AND MEASURES:**

Mortality, functional status (Modified Barthel Index), cognition (Mini-Mental State Examination), and anxiety and depression (Generalized Anxiety Disorder-7, Patient Health Questionnaire-9).

**RESULTS:**

Among 62 participants (mean age 41 ± 16 yr; 50% male), 90-day mortality was 12.9%. By day 90, only 48.4% achieved full functional independence, while 36.8% remained impaired. Normal cognition increased from 22.6% at discharge to 51.6% at follow-up. Notably, 30.6% of survivors reported severe anxiety or depression.

**CONCLUSIONS AND RELEVANCE:**

ICU survivorship in this low-resource setting is marked by substantial persistent morbidity. These findings underscore critical gaps in post-ICU care and the urgent need for scalable, context-appropriate survivorship strategies.

**KEY POINTS**

**Question**: What are the short-term outcomes of ICU survivors in a low-resource setting?

**Findings**: In this prospective cohort study, one-third of ICU survivors experienced persistent physical, cognitive, or psychologic impairment 90 days after discharge.

**Meaning**: Post-ICU morbidity is common in low-resource settings and highlights the need for feasible follow-up and rehabilitation strategies.

Survivorship after critical illness is increasingly recognized as a major contributor to long-term morbidity. Many ICU survivors experience persistent physical disability, cognitive impairment, and psychologic sequelae—collectively described as post-intensive care syndrome—that adversely affect functional independence and reintegration into society (1–4). Although these outcomes are well described in high-income countries, data from low- and middle-income settings remain limited despite a growing burden of critical illness (5–7). In sub-Saharan Africa, research has largely focused on in-hospital mortality, with little attention to multidimensional recovery after discharge (8–10). Rwanda has expanded critical care capacity in recent years; however, structured post-ICU follow-up and rehabilitation services remain scarce. We therefore conducted a prospective cohort study at a national referral ICU in Rwanda to characterize 90-day mortality and physical, cognitive, and psychologic outcomes after ICU discharge using validated assessment tools.

## METHODS

We conducted a prospective cohort study at Rwanda Military Teaching Hospital, a national referral center in Kigali. Consecutive adults (≥ 18 yr) discharged alive from the ICU between March 2025 and September 2025 were enrolled. Patients transferred directly to another facility or who declined consent were excluded. The study was approved by the institutional review board, and written informed consent was obtained. Baseline demographic and clinical data were recorded at ICU discharge. Participants were followed at 7, 30, and 90 days through in-person or telephone assessments. Outcomes included mortality and multidimensional recovery, assessed using validated instruments: the Modified Barthel Index for functional status, the Mini-Mental State Examination for cognition, and standardized screening tools for anxiety and depression. Categorical variables are presented as frequencies and percentages, and continuous variables as means with sds or medians with interquartile ranges, as appropriate. Analyses were performed using Stata, Version 17 (StataCorp, College Station, TX).

## RESULTS

**Figure 1.**
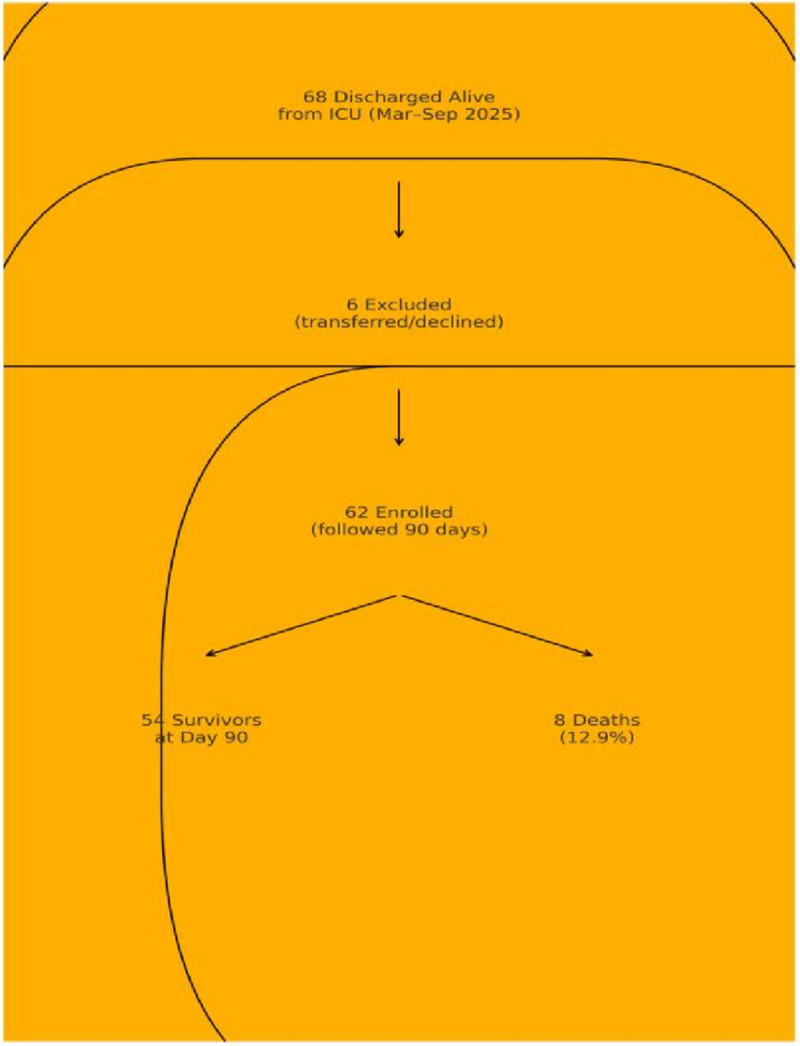
Study flow diagram of ICU survivors enrolled and followed to 90 d.

**Figure 2.**
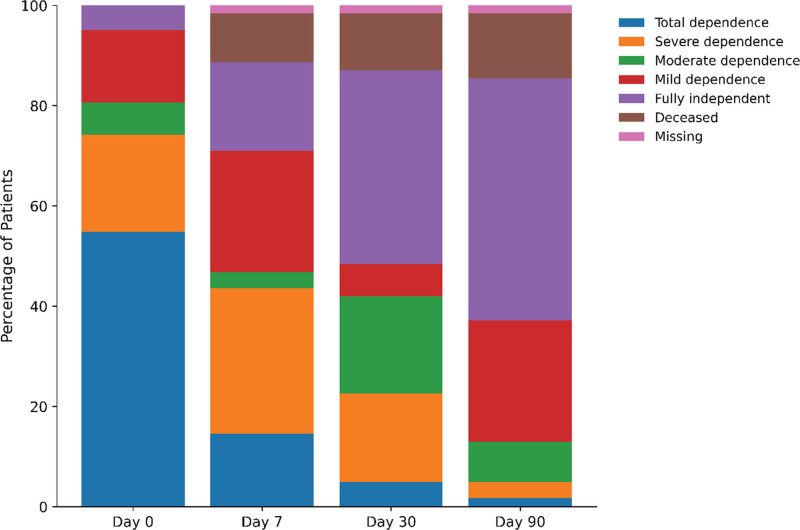
Distribution of functional status from ICU discharge to 90 d (*n* = 62).

During the study period, 68 patients were discharged alive from the ICU; 62 (91.2%) were enrolled and completed 90-day follow-up (**Fig. 1**). The cohort had a mean age of 41 ± 16 years, and 50% were male (**Table 1**). Eight patients died (12.9%) within 90 days after ICU discharge. At ICU discharge, functional impairment was substantial: 54.8% were totally dependent (Modified Barthel Index 0–20). By day 90, 48.4% had regained full functional independence, whereas 36.8% remained functionally impaired. Cognitive recovery improved over time, with normal Mini-Mental State Examination scores increasing from 22.6% at discharge to 51.6% at day 90 (**Fig. 2**). Psychologic morbidity remained common. At ICU discharge, 71.0% of patients had severe anxiety symptoms. At 90 days, 30.6% of survivors continued to report severe anxiety or depression.

**TABLE 1.** Baseline Demographics and Clinical Characteristics of ICU Survivors (*n* = 62)

Characteristic	Value
Age, yr, mean ± sd	41 ± 16
Male sex, *n* (%)	31 (50)
Rural residence, *n* (%)	50 (81)
Any comorbidity, *n* (%)	11 (18)
Primary diagnosis, *n* (%)	
Neurologic (traumatic brain injury, tumors, other CNS)	24 (38.7)
Cardiovascular	7 (11.3)
Respiratory	6 (9.7)
Abdominal surgical	6 (9.7)
Infectious	5 (8.1)
Metabolic/endocrine	4 (6.5)
Maternal complications	6 (9.7)
ICU length of stay, *n* (%)	
1–5 d	28 (45.2)
6–15 d	14 (22.6)
16–30 d	9 (14.5)
31–60 d	10 (16.1)
> 60 d	1 (1.6)

## DISCUSSION

In this prospective cohort from a low-resource setting, substantial multidimensional morbidity persisted during the first 90 days after ICU discharge. Despite a relatively young population with limited documented comorbidity, more than one-third of survivors remained functionally impaired, and nearly one-third reported severe anxiety or depressive symptoms at follow-up. These findings are consistent with reports from high-income settings describing long-term physical, cognitive, and psychologic sequelae after critical illness (1–4). The magnitude of post-ICU morbidity observed was comparable to that reported in high-income settings (4, 5), yet occurred in a context without structured rehabilitation or ICU follow-up services. Data from low- and middle-income countries remain scarce (6–8), and most prior work in sub-Saharan Africa has focused primarily on short-term mortality (9, 10). These findings suggest that survival alone may underestimate the true burden of critical illness in resource-limited health systems. Functional and cognitive recovery improved over time, consistent with prior longitudinal studies of ICU survivors (2, 4). Persistent psychologic symptoms were common, highlighting the need to integrate mental health screening and basic rehabilitation strategies into routine post-ICU care. This study is limited by its single-center design, modest sample size, and 90-day follow-up. Nonetheless, the prospective design and longitudinal use of validated instruments provide important insight into recovery trajectories in a setting where such data are limited. Scalable, context-appropriate survivorship strategies are urgently needed to address the growing population of ICU survivors in low-resource environments (8–10).

## CONCLUSIONS

ICU survivors in this low-resource setting experienced substantial post-discharge morbidity in the absence of structured follow-up care. These findings highlight ICU survivorship as an essential component of critical care delivery and support the need for feasible post-ICU care strategies tailored to resource-limited health systems.
